# Molecular Modeling of Disease Causing Mutations in Domain C1 of cMyBP-C

**DOI:** 10.1371/journal.pone.0059206

**Published:** 2013-03-19

**Authors:** Poornima Gajendrarao, Navaneethakrishnan Krishnamoorthy, Heba Sh Kassem, Sarah Moharem-Elgamal, Franco Cecchi, Iacopo Olivotto, Magdi H. Yacoub

**Affiliations:** 1 Qatar Cardiovascular Research Center, Qatar Foundation, Doha, Qatar; 2 BA-HCM National Programme at Aswan Heart Centre, Egypt; 3 Pathology Department and Clinical Genomics Centre, Alexandria Faculty of Medicine, Alexandria, Egypt; 4 National Heart Institute, Giza, Egypt; 5 Referral Center for Myocardial Diseases, Careggi University Hospital, Florence, Italy; 6 National Heart and Lung Institute, Imperial College London, United Kingdom; National Institutes of Health, United States of America

## Abstract

Cardiac myosin binding protein-C (cMyBP-C) is a multi-domain (C0–C10) protein that regulates heart muscle contraction through interaction with myosin, actin and other sarcomeric proteins. Several mutations of this protein cause familial hypertrophic cardiomyopathy (HCM). Domain C1 of cMyBP-C plays a central role in protein interactions with actin and myosin. Here, we studied structure-function relationship of three disease causing mutations, Arg177His, Ala216Thr and Glu258Lys of the domain C1 using computational biology techniques with its available X-ray crystal structure. The results suggest that each mutation could affect structural properties of the domain C1, and hence it’s structural integrity through modifying intra-molecular arrangements in a distinct mode. The mutations also change surface charge distributions, which could impact the binding of C1 with other sarcomeric proteins thereby affecting contractile function. These structural consequences of the C1 mutants could be valuable to understand the molecular mechanisms for the disease.

## Introduction

Hypertrophic cardiomyopathy (HCM) is an inherited heart disease that has continued to interest and intrigue clinicians, molecular biologists, biochemists and modellers [1]–[4]. HCM is usually caused by mutations in the genes encoding for sarcomeric proteins [5]–[8]. To date, more than 800 mutations have been reported in the genes that encode for sarcomeric proteins [9], [10]. However, the mechanism by which a gene mutation can cause such a massive difference in the phenotype and its function remains largely unknown. Therefore, the objective of this study is to investigate the basic mechanism by which the mutations translate to the phenotype.

The sarcomere is the basic unit of a muscle and it is composed of a variety of proteins including myosin and actin as the major components that form thick and thin filaments, respectively. Other accessory proteins such as myosin binding protein-c (MyBP-C) [11], titin, troponin, tropomyosin etc. take part in maintaining structure and regulating function of the sarcomere.

A mutational analysis study on an Egyptian cohort through Bibliotheca Alexandrina HCM (BA-HCM), a national programme showed that mutations in cardiac MyBP-C (cMyBP-C) gene are a common cause of HCM in Egypt [12]. More than 200 disease causing mutations have been reported in the cMyBP-C [7], [13]–[19].

cMyBP-C is a large sarcomeric protein with multiple domains and a component of thick filaments [20]–[22]. It is solely expressed in the heart of mammals [23], [24]. The structure of cMyBP-C is composed of 11 domains including eight immunoglobuline(Ig)-like domains and three fibronectin(Fn)-like domains, which are termed as C0–C10 [25]. Each domain is a globular protein. The three-dimensional structure is available for only a few of the domains including C0, C1, C2 and C5 [26]–[30]. The N-terminus of the protein including domains C0, C1, cMyBP-C motif and C2 plays a significant role in the regulation of interaction with myosin [31], [32] and/or actin [33], [34]. Specifically, the domain C1 has been found to interact with sub-fragment 2 of myosin [26]. In addition, N-terminal of this domain might bind with actin through Pro-Ala rich region [27].

**Figure 1:**
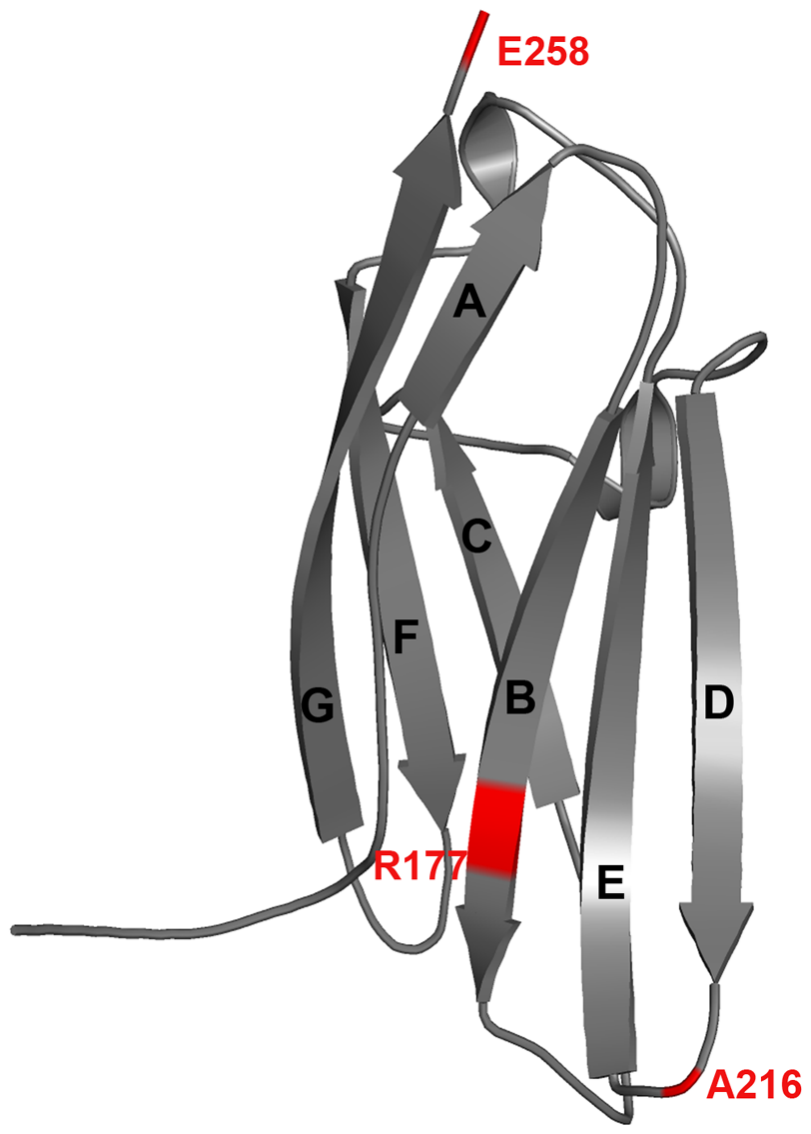
Structural features of the domain C1 of cMyBP-C. Positions of the examined HCM causing mutations are shown in red. The C1 consists of seven β-stands that form two β-sheets, where sheet-1 consists of strands A, C, F and G while sheet-2 comprises strands B, D and E both with anti-parallel packing of adjacent strands. Refer Figure 4 and 6 for the key residues.

Here, we studied three disease causing missense mutations of the domain C1 that were recently identified in Egypt for the first time by Kassem et al., [12]. These HCM causing mutations: (i) Arg177His (ii) Ala216Thr and (iii) Glu258Lys are located in the strand-B, the D/E loop and the C-terminal of strand-G, respectively (Figure 1). These mutations have also been reported by others in different parts of the world [35]–[37]. In particular, Glu258Lys has been reported to cause a founder effect in Italian population [38]. Clinical studies suggest the effect of mutations (ie., contractile dysfunction [7], [8], [12], [15], [18], [36]–[38]), yet less is known about their structure-function relationship. Although the structural positions of the mutations are known [26]–[28], their exact role in causing HCM is unclear. Computational biology might prove as a valuable tool at the molecular level. These include molecular dynamics (MD) simulations, Floppy Inclusions and Rigid Substructure Topography (FIRST) [39], and electrostatic potential calculations. Here, we have used these techniques to analyse the mutation-induced changes in the structural and electrostatic properties that could alter the function of the C1 and cMyBP-C, and might therefore lead to the disease.

## Materials and Methods

### Model Repair

The high resolution crystal structure of domain C1 of cMyBP-C (PDB ID: 2V6H, 1.55 Å) [27] was taken from the protein data bank (PDB, http://www.rcsb.org/pdb/) for our study. The C1 consists of seven β-stands that form two β-sheets, where sheet-1 consists of strands A, C, F and G while sheet-2 comprises strands B, D and E, both with anti-parallel packing of adjacent strands (Figure 1). The structure of domain C1 has four missing residues (Ala181-Leu184) in the B/C loop. The Discovery Studio V3.1 (DS) was used to construct the missing residues (Accelrys, San Diego, USA) [40] and the resulting model was subjected to energy minimization using Groningen Machine for Chemical Simulation (GROMACS) V4.5.4. The energy minimized C1 structure was used to build the mutants that were modelled using the DS “build and edit protein”. A total of four MD simulations were carried out including one wild type and three mutant C1 structures with production runs of 10 ns.

### MD Simulations of Domain C1 of cMyBP-C

Energy minimization was carried out for the domain C1 using the steepest descent algorithm with a tolerance of 2000 kJ/mol/nm using the GROMACS simulation package [41], [42]. The energy minimized structure was used as the starting structure for the MD simulations. The GROMOS96 [43] force field was applied to the C1 structure while the SPC3 [44] water model was used to create the aqueous environment. The protein was solvated in a cubic box with the size of 0.8 nm. Periodic boundary conditions were applied in all directions and the system was neutralized by adding Na^+^ ions. The resulting systems contain ∼30860 atoms. A twin range cut-off was used for long-range interactions: 0.8 nm for van der Waals interactions and 1.4 nm for electrostatic interactions. All bond lengths were constrained with the LINCS [45] algorithm. The SETTLE [46] algorithm was applied to constrain the geometry of the water molecules. The energy minimized system was subjected to 100 ps equilibration. This pre-equilibrated system was subsequently subjected to 10 ns of production MD simulations with a time-step of 2 fs at constant temperature (300 K), pressure (1 atm) and number of particles, without any position restraints [47]. The snapshots were collected at every 10 ps. The trajectories were analyzed using GROMACS analysis tools and the structures were analyzed using DS and PyMOL (www.pymol.org).

### Rigidity Analysis

The program FIRST is used to identify rigid and flexible regions of the C1 network graphs. In FIRST, the cut-off parameters for the energy and the hydrophobic interactions were set to −0.7 and 1.0, respectively. Structural degrees of freedom, intra-molecular interactions and number of rigid clusters were also calculated.

### Electrostatic Surface Calculation

The electrostatic potential was calculated for the WT as well as the mutants using the Delphi package provided in the DS. The Delphi charges were assigned for the structures and the surface electrostatic potential map was obtained by solving the Poisson-Boltzmann equation.

## Results

### Trajectory-based Analyses for the MD Simulated Systems

**Figure 2:**
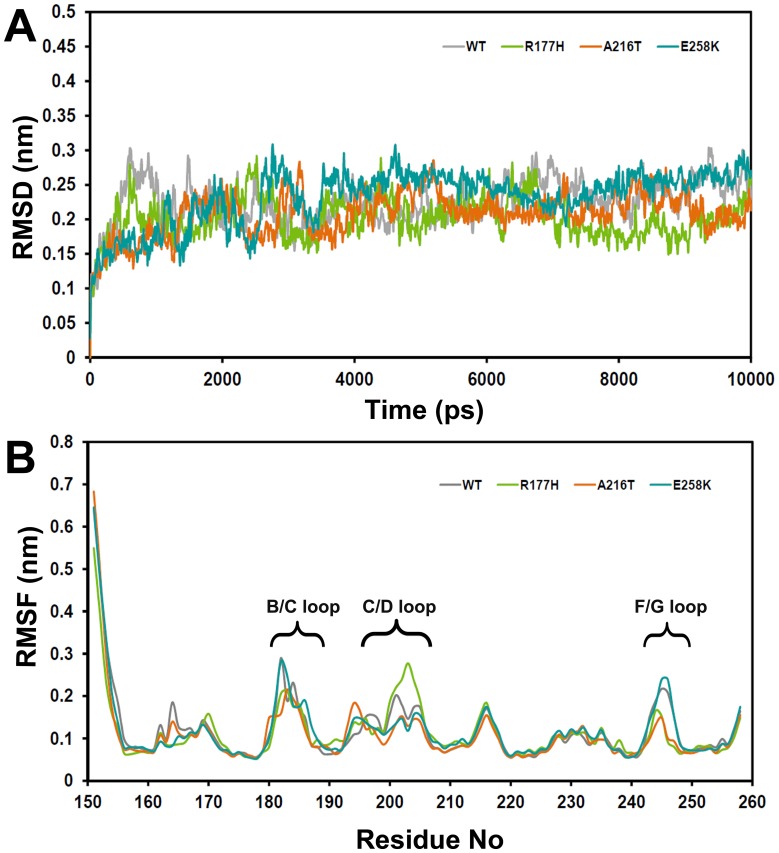
Trajectory-based structural stability analyses of WT and mutants. (A) Root mean square deviation (RMSD) and (B) average root mean square fluctuation (RMSF) of residues during MD simulations.

In order to find out the structural stability of WT and mutants of the C1 dynamically, root mean square deviation (RMSD) was computed with respect to their initial structures for the Cα-atoms (Figure 2A) throughout the MD simulations. It showed that the deviation pattern was different for WT and mutants. However, after the equilibration phase, all the systems stayed within ∼0.3 nm and this suggested that the structures remain stable.

To determine the flexible regions of the systems, the average root mean square fluctuations (RMSF) were calculated for 10 ns of the MD simulations and generated as a 2D plot with respect to the Cα-atoms of the residues (Figure 2B). Here, residues that are fluctuated more than 0.2 nm considered as flexible regions. As expected, the long loop at the N-terminal and the loops B/C, C/D and F/G were observed as flexible regions while the rest of the domain remained rigid.

These trajectory-based analyses have not provided significant differences in the behaviour of the systems. Hence, we tried to examine the individual structures in detail to see whether the mutations induced any changes in the secondary structural elements during the simulations. For this purpose, every one nanosecond structures were collected, observed and analysed for 10 ns.

### Secondary Structural Changes and Interactions of the Mutated Residues

**Figure 3:**
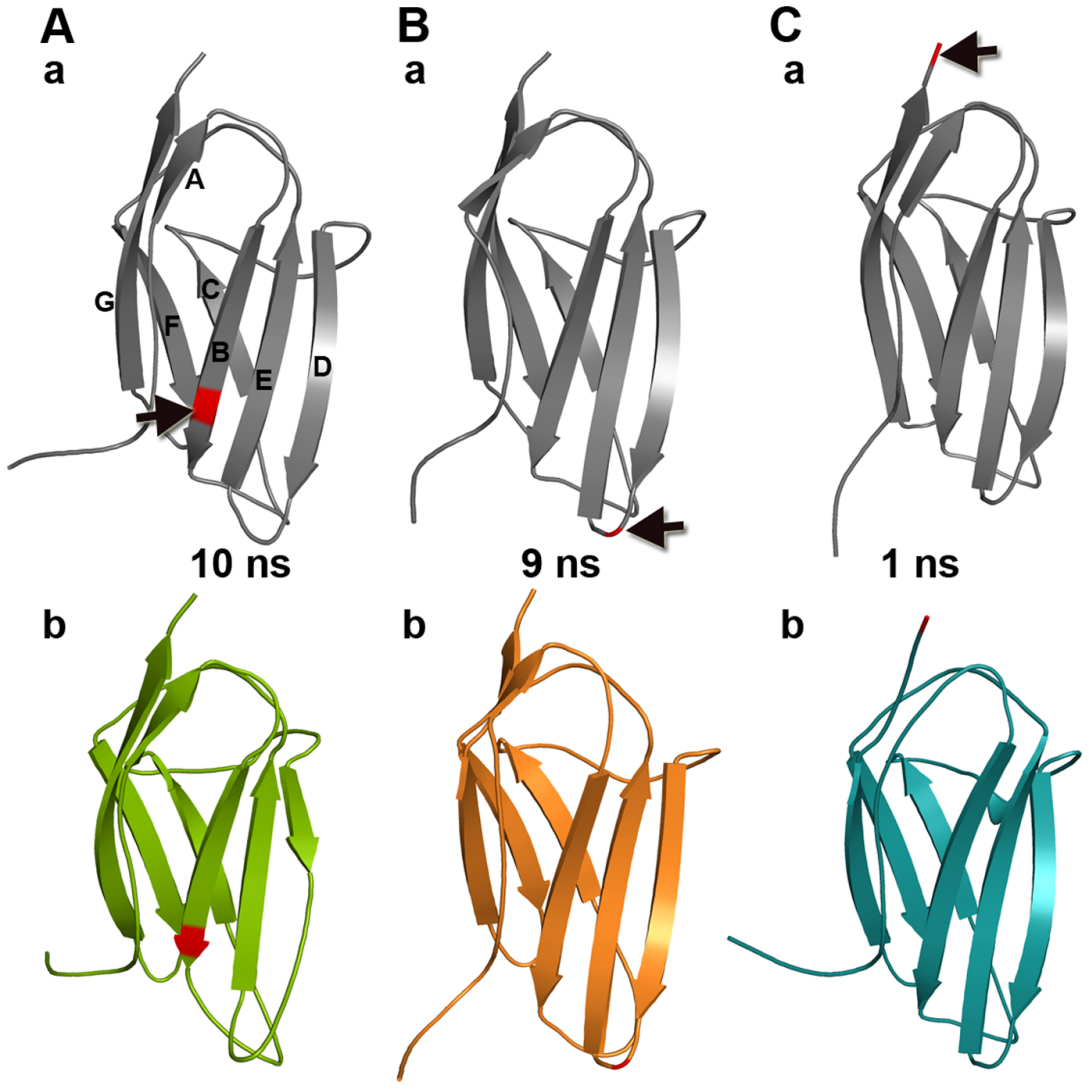
Secondary structural changes. The representative snapshots of WT and mutants are shown. (A) a) WT and b) Arg177His at 10 ns, (B) a) WT and b) Ala216Thr at 9 ns and (C) a) WT and b) Glu258Lys at 1 ns. In the structures, red color cartoon and black arrows indicate position of the mutation.

The nature of the secondary structural elements in the WT (core structure) has been conserved during the MD simulations (Figure 3Aa, 3Ba and 3Ca). However, all three mutants displayed changes in their secondary structural elements at several stages of the MD simulations and the structures that showed major changes are represented in Figure 3Ab, 3Bb and 3Cb. Lose of secondary structural changes in the strand A (Fig. 3Bb and 3Cb) was not considered in the analysis, as it might be due to the highly flexible long N-terminal loop (Fig. 2B).

Here, we studied interactions of the residues at positions 177/216/258 with their neighbouring residues in the WT and mutants in order to investigate whether behaviour of the mutants might be the cause for the observed structural changes. This has been performed by identifying two layers of neighbouring residues which are connected via hydrogen bonds. Here, the residues which interact directly with the residue at 177/216/258 are called as first layer residues and the residues that are (indirectly) linked with 177/216/258 through a mediator that is via the first layer residues are considered as the second layer of residues.

**Figure 4:**
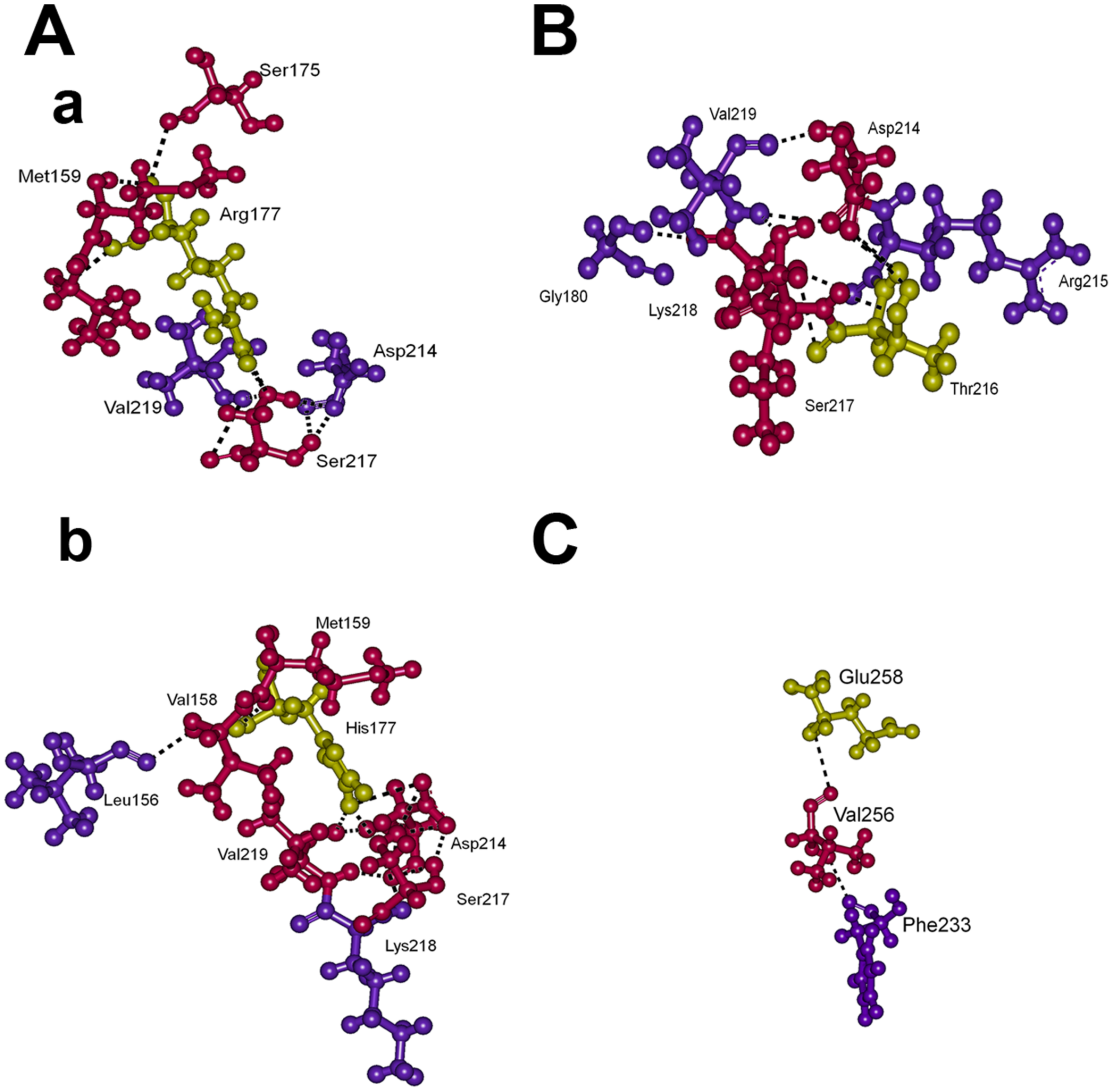
Intra-molecular interactions. For the represented snapshots of MD simulations (Figure 3), two layers of neighbouring residues between corresponding WT and mutant residues along with their hydrogen bonds are displayed. Here, (A) a) WT and b) Arg177His at 10 ns, (B) Ala216Thr at 9 ns and (C) WT at 1 ns. At 9 ns Ala216 of WT and at 1 ns Lys258 of Glu258Lys did not make any interactions, hence they are not shown. WT and mutant residues 177, 216 and 258 are shown in yellow, and the residues directly interacting with these residues are shown in pink and are described as first layer. The residues that are interacting with first layer of residues are depicted as second layer and shown in violet.

At 10 ns of the Arg177His mutant simulation, a major part of the β-strand D has been converted into loop that extend the D/E loop (Figure 3Ab). This structural change occurred in the vicinity of the strand B, where the relevant mutation is positioned. Here, we monitored interactions between the residue 177 and its neighbouring residues. In the WT, Arg177 directly interacts with Met159, Ser175 and Ser217 and these residues further contact Asp214 and Val219 (Figure 4Aa). Whereas, in the mutant, His177 forms hydrogen bonds with Val158, Met159, Asp214, Ser217 and Val219 at the first layer and the network extends further through Leu156 and Lys218 (Figure 4Ab).

The mapping of intra-molecular interactions associated with the residue 177 shows that the network based on Arg177 is smaller than the one based on His177. It is mainly because of the exposed side-chain of the arginine that was not involved in to form many interactions in WT, whereas, the ring structure of His177 fits well within the interaction pocket to make many interactions. The increase in the number of hydrogen bonds based on His177 at this region introduce more bonding constrains that could affect its local flexibility.

At 9 ns of the simulation of the mutant Ala216Thr, the length of the F helix has been increased and the long strand G has been divided into two strands, while the mutation spot is located at the loop D/E (Figure 3Bb). The native residue alanine at 216 makes no contacts because it is exposed to the surface from the loop D/E and due to its short side-chain. On the other hand, the long side-chained hydrophilic residue threonine at 216 forms hydrogen bonds with Asp214, Ser217 and Lys218 and they continue the network with Gly180 and Val219 (Figure 4B).

Although the interaction pattern has been altered near the loop D/E, the structural change has occurred at the F-G interface. It suggests that the impact of this mutation could be at the distal regions of the domain C1 (Figure 3Bb) where some of the interactions are modified. This behaviour is different from the previous mutation studied above (Figure 3Ab).

At 1 ns of the mutant Glu258Lys, half of the long strand-G has been converted into a loop. The mutation spot is located at the C-terminal region of this strand. The Glu258 in the WT directly interacts with Val256 of the same strand-G and indirectly with Phe233 of the loop E/F via Val256, while, Lys258 has no interactions with its nearby residues (Figure 4C). This mutation has similar behaviour as the Arg177His, which affects the neighbouring regions indirectly.

**Figure 5:**
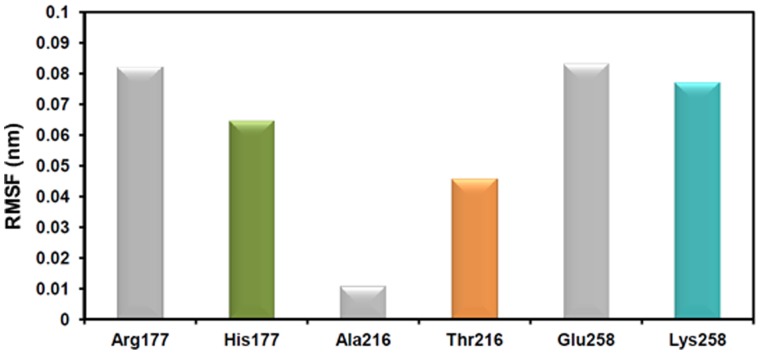
Fluctuations of the WT and mutant residues during MD simulations. The average RMSF of the mutated residues for 10 ns are plotted in 2D bar graph. The bars in gray and in different colours (green, orange and aqua green) represent the WT and the mutants, respectively.

The mapping of neighbouring contacts shows the local hydrogen bonding networks that are associated with the residues at the mutational positions. However, for all three mutations, it indicates that they are not directly involved in triggering the secondary structural changes. Thus, to relate these changes to the secondary structural changes, we analysed the fluctuations of the mutated residues during the MD simulations. The average RMSF (Figure 5) for 10 ns of the systems shows different behaviour for the WT and the mutated residues at their corresponding positions. The arginine at position 177 fluctuates higher than the histidine at the same position and a similar behaviour was observed for Glu258Lys, where glutamic acid fluctuates slightly higher than lysine. On the other hand, the mutated residue thronine fluctuates significantly more than the native alanine. These results imply that modifying the long side-chain residues (Arg) to short (His) and vice versa (Ala, to Thr and Glu to Lys) at key positions could make them rigid or flexible, respectively. This might induce a local structural instability and intervene with the native bonding of neighbouring residues which can induce structural changes.

### Rigidity and Flexibility of Residues Linked with Structural Changes

**Figure 6:**
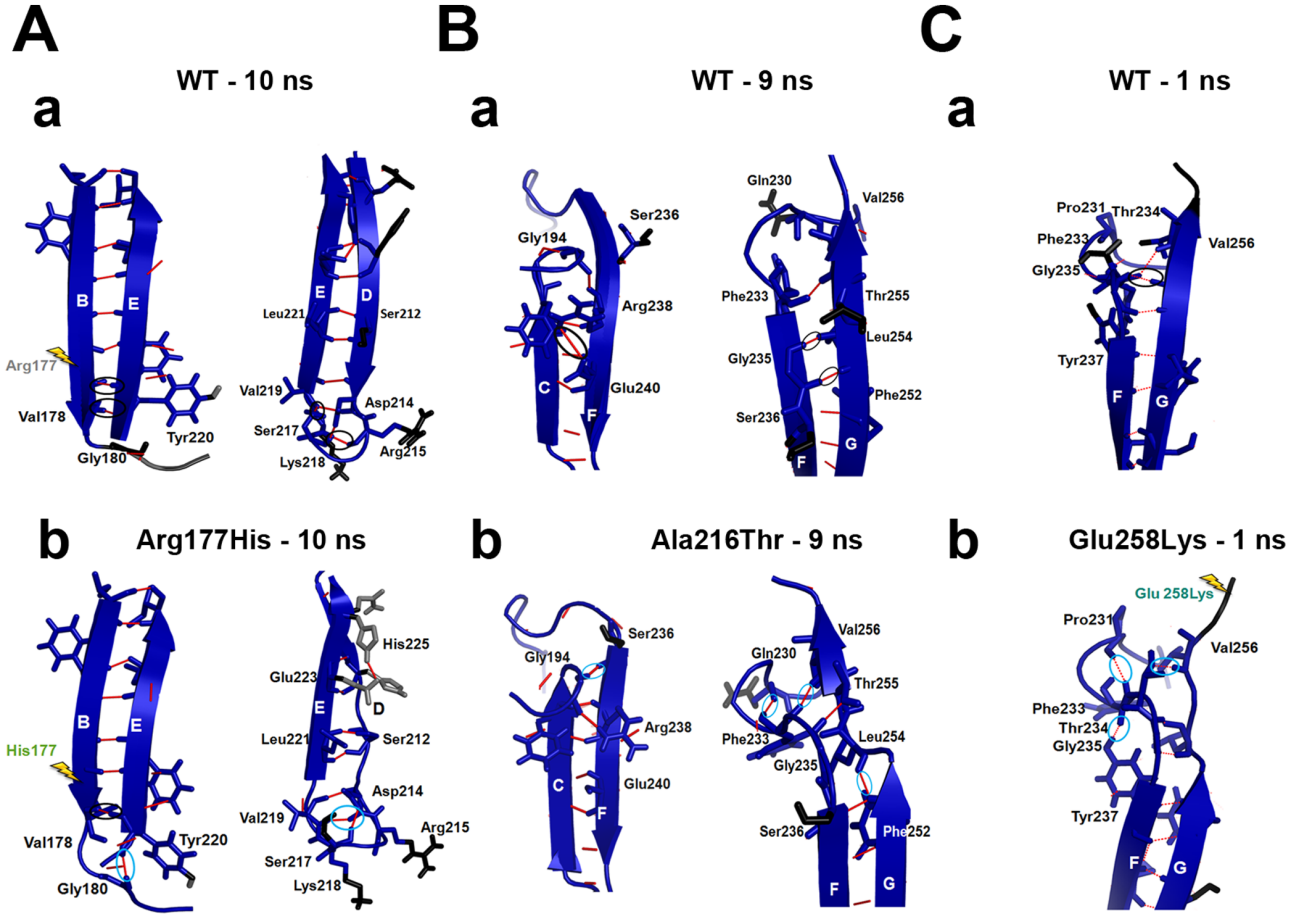
Molecular explanations for the structural changes. The rigidity analysis provided information based on the structural rigidity and flexibility of residues. (A) 10 ns snapshots of a) WT and b) Arg177His, (B) 9 ns snapshots of a) WT and b) Ala216Thr and (C) 1 ns snapshots of a) WT and b) Glu258Lys. Here, blue represents rigid regions and black and gray indicates flexible regions. The mutational spots are shown in yellow arrow. As the Ala216Thr affected the distal region of the domain, the position of this mutation has not shown. The hydrogen bonds (red lines) that are missing in the mutants are marked in black and the newly formed hydrogen bonds in the mutants are shown in light blue.

The identification of changes in rigid and flexible regions in the WT and mutated systems of C1 can provide a molecular explanation for the secondary structural changes at the affected regions. FIRST is a program that identifies rigidity and flexibility of substructures in a macromolecule and offers details related to intra-molecular interactions. Therefore, we used FIRST to analyse the representative 3D structures to monitor the key hydrogen bonds, which normally determine the rigidity, and the residues that are involved in the formation of β-sheets. We focused our analyses towards the structurally modified regions of the β-sheets (Figure 6), which could affect the rigidity of the rest of the domain that are structurally conserved.

The structural architecture of a typical antiparallel β-sheet is important to maintain integrity of domain C1 and other Ig-like domains of cMyBP-C [26]–[30]. These types of sheets are constructed with antiparallel β-strands and the majority of the building blocks are hydrophobic residues. The β-sheet is formed through making hydrogen bonds between carbonyl oxygen of one strand and amino hydrogen atom of the other strand. Thus, each residue from the strand-1 makes two hydrogen bonds with their neighbours on the strand-2. This bonding pattern is required for the formation of a successful β-sheet. Interfering with any of the residues in the sheet via an inappropriate mutation will not only break the continuity of the bonding pattern but also make the side-chain of the mutated residue flexible, which could disrupt the natural bonding of neighbours and might result in loss of rigidity or secondary structural elements.

In the WT and the Arg177His at 10 ns, we focused on the sheet-2 as it is the structurally modified region due to the mutation. Here, the sheet is shown into two interfaces, the B-E and D-E interfaces (Figure 6Aa–b). The secondary structural change in the B-E interface was observed at the C-terminal of the strand B and D. At this region, the sheet was maintained with two hydrogen bonds between Val178 and Tyr220 in the WT. However, one of the hydrogen bonds between these two residues was lost in the mutant which resulted in the side-chain movement of Tyr220. This side-chain movement of Tyr220 now forms another hydrogen bond with Gly180, which is also flexible as it is in the B/C loop. The movement of the aromatic side-chain of Tyr220 appears to affect the bonding pattern in its vicinity especially at the D-E interface of Arg177His. Although the Ser212 forms two hydrogen bonds with Leu221 in both WT and Arg177His, in the latter case one of the hydrogen bonds is not from the backbone atom. This change together with the motion of Tyr220 might affect the remaining β-strand D and cause rearrangement in the pattern of hydrogen bonds such as loss of hydrogen bonds between Ser217 ↔ Val219 and Arg215 ↔ Lys218 and formation of new hydrogen bonds between Asp214 ↔ Ser217 and Asp214 ↔ Val219 (Figure 6Ab) (↔ indicates a single hydrogen bond). These changes might have caused the loss of secondary structure in the D–E interface.

These results suggest that replacement of Arg177 to histidine initiates the changes by disturbing the normal bonding of neighbour Val178 with Tyr220. This tyrosine becomes flexible and interrupts the usual bonding of its neighbour Leu221 with Ser212 and rearranges the pattern of hydrogen bonds of the other mentioned pairs and thus could bring structural change at strand B, D and E.

The next mutation (Ala216Thr) is located on the loop D/E in the sheet-2, which affected the distal region at the F–G interface in the sheet-1. In this case, Arg238 of WT formed a strong charge based interaction with Glu240, which was missing in the mutant Ala216Thr and Gly194 formed a new interaction with Ser236 at the C-F interface (Figure 6Ba–b). While in the F–G interface, Gly235 of WT formed two hydrogen bonds with Leu254. However, the backbone interactions were lacking in the mutant between Gly235 and Leu254 rather they gained one of their lost interactions through contacting Thr255 and Phe252, respectively. This rearrangement of hydrogen bonds induced breakage of the strand G in Ala216Thr, this change brought the E/F loop closer and helped in the establishment of new hydrogen bonds between Phe233 ↔ Gln230. Moreover, this type of change played a role in rearrangement of hydrogen bonds at the F/G loop region (data not shown) such as loss of three hydrogen bonds between Phe247 ↔ Lys246 and Lys246 ⇔ Asp248 and formation of hydrogen bonds between Thr243 ⇔ Lys246 and Lys246 ↔ Asp245 (Figure 6Bb, ⇔ two hydrogen bonds).

The observed changes in this mutation occurred at the mid of the F–G interface. Although this region is away from the mutational spot on the D/E loop, these two regions are linked by the strand E and the E/F loop. The changes are possibly initiated by the Gly235 (and Thr234), which affected its normal interactions with Leu254 and thereby modified the usual bonding pattern of several residues in the vicinity. Thus, it indicates that the mutation at the other end of the segment in the C1 at some point could induce the structural changes through the strand E–E/F loop.

In the Glu258Lys at 1 ns, the sheet-1 has been affected. Here, at 1 ns of WT in the F–G interface, Val256 forms a hydrogen bond with Phe233 (Figure 6Ca–b). This hydrogen bond is missing in the mutant. In addition, new hydrogen bonds were observed between Pro231 ↔ Thr234, Phe233 ↔ Tyr237, Thr234 ↔ Val256. This rearrangement (loss and gain) in the hydrogen bonding pattern could have affected the structure locally (the G-strand).

Here, the observed changes suggest that mutating a small negatively charged residue Glu258 into a long side-chained positively charged lysine at the C-terminal of the strand G can initiate the structural changes through its neighbouring region. It might have started to intervene with the bonding of Val 256 with Phe233. This was not observed in the mutant. This change has affected the bonding pattern of its neighbours and modified usual network of several residues nearby and could have transformed the long strand into two strands divided by a loop.

The rigidity analysis together with RMSF results suggest that the fluctuations of the three mutated residues can cause indirect effects on the rigidity of the domain by breaking/making of interactions with its neighbouring residues thus modifying secondary structural elements.

### Mutational Impact on the Electrostatic Surface of the Systems

The change in the internal molecular architecture can alter the surface charge distribution, which is important for protein-protein interactions. It is significant for this study as C1 in cMyBP-C interacts with myosin and actin using surface electrostatic properties [26], [27].

**Figure 7:**
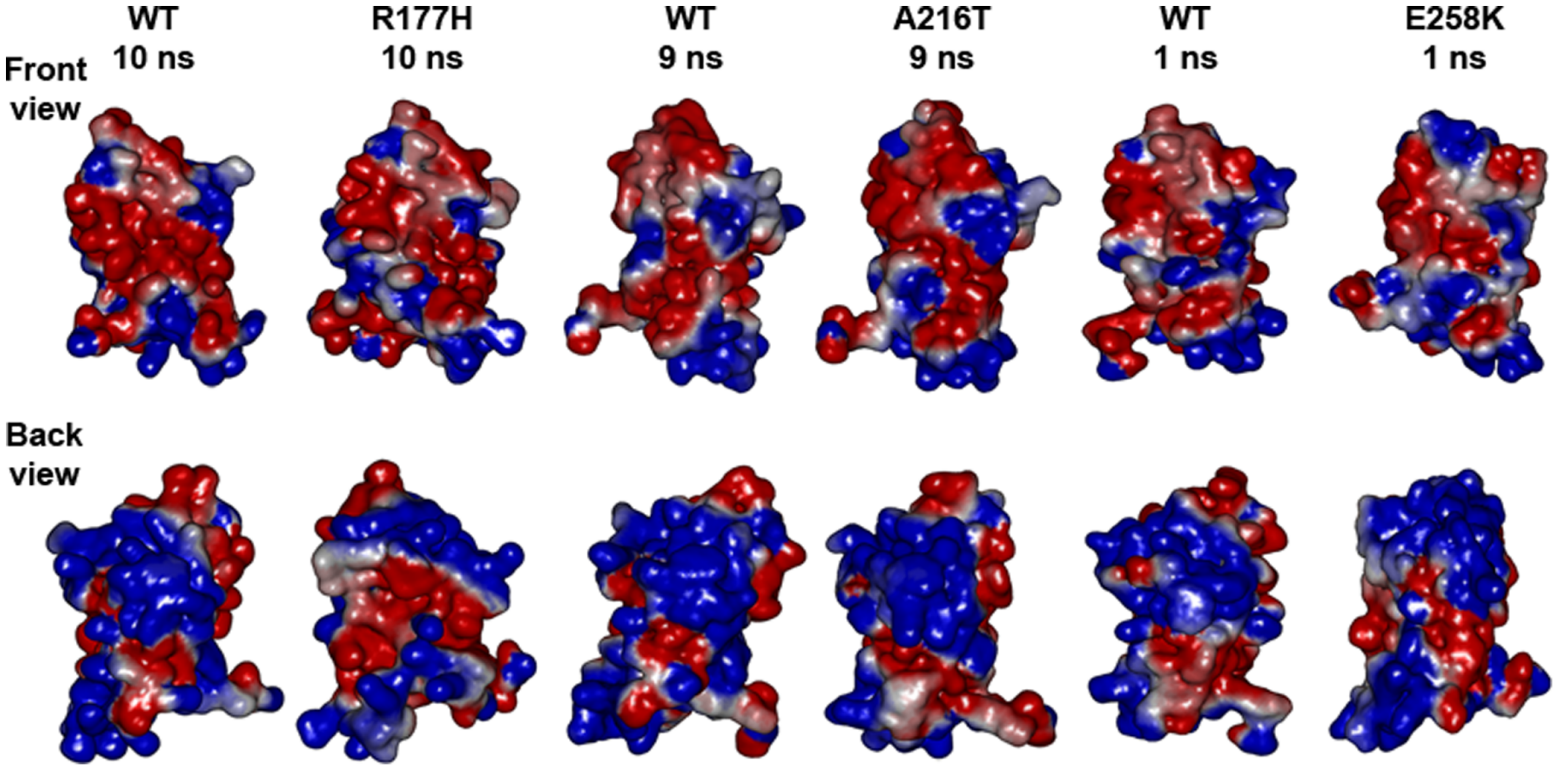
Electrostatic surface potential map. Front view and back view of surface electrostatic potential map for the snapshots shown in Figure 3. Where, blue, red and white represent positive, negative and hydrophobic electrostatic potential, respectively. WT at 10 ns, 9 ns and 1 ns were in different conformational states due to the simulations that were performed after the equilibration. In addition, the N-terminal of the protein was highly flexible during all 4 simulations (Figure 2b), hence the electrostatic surface map of the representative snapshots shows considerable change at their N-terminal.

Here, the results of the electrostatic calculations (Figure 7 WT and Arg177His at 10 ns) showed that His177 with the help of Leu156, Val158 and Met159 pushes Arg160 near the mutated region to introduce a positive patch in between the negative surface. On the other side, the changes at the sheet-2 bring down Lys185 and Lys190 through the B/C loop to create space for a negative surface created by Glu240 and Asp198.

The Thr216 interaction with Asp214 allows Arg160 and Arg177 to project a positive surface near the strand B. Furthermore, the change in the distal region at the N-terminal loop affects the neighbour loop F/G and brings the hydrophobic surface through Phe247 (Figure 7, WT and Ala216Thr at 9 ns).

The position of 258, which is highly conserved with negatively charged glutamic acid in cMyBP-C for most of the organisms, was replaced with a positively charged lysine residue in the mutant Glu258Lys. Hence, the presence of Lys258 is responsible for the formation of a positive patch at the C-terminal in this mutant at both the front and back view of the 1 ns snapshots (Figure 7 WT and Glu258Lys).

The structural basis of the interactions in the domain C1 depends on conformational integrity and surface charge distributions. Here, results from our study reveal how the mutations in a single domain can initiate structural changes by altering the intra-molecular interactions. These internal changes could bring local structural constrains and affect the formation of secondary structural elements. Consequently, it influences the domain conformation and modifies the nature of surface charge distribution, which is important for this domain to interact with neighbouring domains and to interact with other sarcomeric proteins such as myosin and actin to regulate muscle contraction.

## Discussion

This study has shown that the disease causing mutations affect the structural properties of domain C1 through changing (i) residue flexibility/rigidity (ii) intra-molecular interactions, (iii) secondary structural elements and (iv) surface electrostatics. As these changes are inter-linked, this chain of modifications might leads to altered molecular mechanism. Arg177His and Glu258Lys have similar patterns such as via affecting its neighbouring regions, sheet-2 and sheet-1, respectively. In contrast, Ala216Thr affects the distal region from the mutational spot of the C1.

The domain C1 is flanked by a structurally and functionally important proline-alanine rich region and the cMyBP-C motif on both sides. The proline-alanine rich region regulates cross-bridge speed thus plays a significant role in the sarcomere [48]. Whereas, the conserved motif is found to be interacting with sub-fragment 2 of myosin [26]. Hence, any structural change in the domain C1 might reflect in the function.

The relationship between structural change and molecular mechanism is fundamental in understanding phenotype [49]. The structural changes observed here in domain C1 could disturb phosphorylation sites on neighbouring motif and thereby influence Ca2+ signalling and energy supply for contractile function, thus producing energy depletion which has been identified as possible mechanism in HCM [8], [50]. Recent reports support the notion that the normal phosphorylation in the motif regulates the Ca2^+^-dependent interactions between cMyBP-C [51] and calmodulin (CaM)/calmodulin kinase II (CaMKII, [52]) and thus dysregulation of calcium handling could be produced by the structural changes reported here, thereby play an important role in the pathophysiology of HCM. Furthermore, the changes in electrostatic and structural properties due to the mutations in C1 could impact neighbouring phosphorylation sites. This is supported by the fact that the removal of cMyBP-C from native cardiac thick filaments slowed down motion generation of actomyosin, which was also modulated by phosphorylation [53]. However, it is unclear that how these types of disease causing mutations are not blocked by the natural quality control systems (mRNA decay, ubiquitin-proteasome system and autophagy), which commonly control their expressions [54]. This opens up new research avenues to explore molecular therapeutic agents that can reinforce the natural way of quality control to prevent this cardiac disease.

In conclusion, the HCM causing mutations studied here potentially changes the native structural properties of the domain C1 of cMyBP-C. We have given possible molecular explanations to understand how the mutations could initiate the malfunction. It has shown that three mutations have different mechanisms to disturb the structural integrity of the domain C1. The molecular changes described here could distract binding of the molecule to actin and/or myosin and thus interfere with both contractile and electrical functions. As cMyBP-C is a major player in functional regulation and structural integrity of the sarcomere, its dysregulation can be responsible for at least some of the pathological changes observed in HCM.
